# Tetra­kis(3,5-xylidinium) dihydrogen cyclo­hexa­phosphate dihydrate

**DOI:** 10.1107/S1600536809054452

**Published:** 2009-12-24

**Authors:** Houda Marouani, Mohamed Rzaigui

**Affiliations:** a Laboratoire de Chimie des Matériaux, Faculté des Sciences de Bizerte, 7021 Zarzouna Bizerte, Tunisia

## Abstract

In the title compound, 4C_8_H_12_N^+^·H_2_P_6_O_18_^4−^·2H_2_O, the complete cyclo­hexa­phosphate anion is generated by inversion symmetry. Crystal cohesion and stability are supported by electrostatic inter­actions which, together with N—H⋯O and O—H⋯O hydrogen bonds, build up a three-dimensional network.

## Related literature

For related structures, see: Khederi *et al.* (2001 ▶); Rayes *et al.* (2004 ▶); Amri *et al.* (2008 ▶); Janiak *et al.* (2000 ▶). For a discussion on hydrogen bonding, see: Brown (1976 ▶). For tetra­hedral distortions, see: Baur (1974 ▶). For the preparation of cyclo­hexa­phospho­ric acid, see: Schülke & Kayser (1985 ▶).
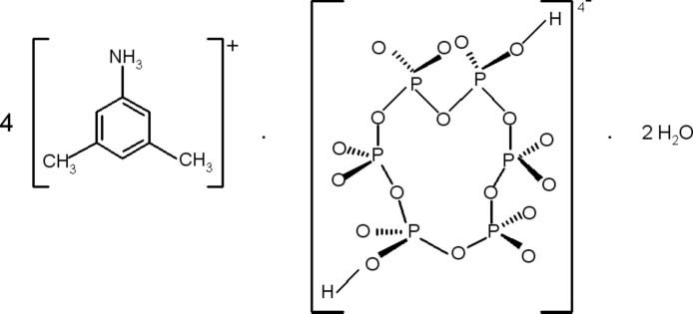


## Experimental

### 

#### Crystal data

4C_8_H_12_N^+^·H_2_P_6_O_18_^4−^·2H_2_O
*M* *_r_* = 1000.61
Monoclinic, 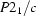

*a* = 17.254 (3) Å
*b* = 11.763 (5) Å
*c* = 11.556 (2) Å
β = 106.41 (3)°
*V* = 2249.9 (11) Å^3^
*Z* = 2
Mo *K*α radiation
μ = 0.32 mm^−1^
*T* = 293 K
0.35 × 0.20 × 0.01 mm

#### Data collection

Enraf–Nonius CAD-4 diffractometer
10097 measured reflections
9844 independent reflections
5567 reflections with *I* > 2σ(*I*)
*R*_int_ = 0.039
2 standard reflections every 120 min  intensity decay: 11%

#### Refinement

*R*[*F* ^2^ > 2σ(*F* ^2^)] = 0.052
*wR*(*F* ^2^) = 0.141
*S* = 1.02
9844 reflections
295 parameters
3 restraints
H atoms treated by a mixture of independent and constrained refinement
Δρ_max_ = 0.50 e Å^−3^
Δρ_min_ = −0.51 e Å^−3^

### 

Data collection: *CAD-4 EXPRESS* (Enraf–Nonius, 1994 ▶); cell refinement: *CAD-4 EXPRESS*; data reduction: *XCAD4* (Harms & Wocadlo, 1995 ▶); program(s) used to solve structure: *SHELXS86* (Sheldrick, 2008 ▶); program(s) used to refine structure: *SHELXL97* (Sheldrick, 2008 ▶); molecular graphics: *ORTEP-3* (Farrugia, 1997 ▶); software used to prepare material for publication: *WinGX* (Farrugia, 1999 ▶).

## Supplementary Material

Crystal structure: contains datablocks I, global. DOI: 10.1107/S1600536809054452/hb5267sup1.cif

Structure factors: contains datablocks I. DOI: 10.1107/S1600536809054452/hb5267Isup2.hkl

Additional supplementary materials:  crystallographic information; 3D view; checkCIF report

## Figures and Tables

**Table 1:** Hydrogen-bond geometry (Å, °)

*D*—H⋯*A*	*D*—H	H⋯*A*	*D*⋯*A*	*D*—H⋯*A*
O8—H8⋯O6^i^	0.82	1.71	2.421 (2)	144
O1*W*—H2*W*⋯O9	0.85 (1)	2.01 (1)	2.831 (2)	164 (2)
O1*W*—H1*W*⋯O5^ii^	0.85 (1)	2.00 (1)	2.829 (2)	165 (2)
N1—H1*A*⋯O9^iii^	0.89	2.03	2.910 (2)	170
N1—H1*B*⋯O1*W*	0.89	1.89	2.769 (2)	169
N1—H1*C*⋯O3	0.89	1.93	2.738 (2)	151
N2—H2*A*⋯O3^i^	0.89	1.94	2.801 (2)	161
N2—H2*B*⋯O2^iv^	0.89	1.97	2.768 (2)	148
N2—H2*C*⋯O5	0.89	1.83	2.719 (2)	175

Symmetry codes: (i) 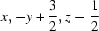; (ii) 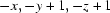; (iii) 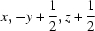; (iv) 
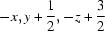
.

**Fig. 1.**
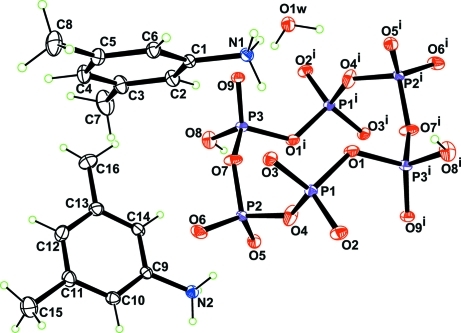
A view of (I) with displacement ellipsoids drawn at the 30% probability level. Symmetry code: (i) - x, - y, - z.

**Fig. 2.**
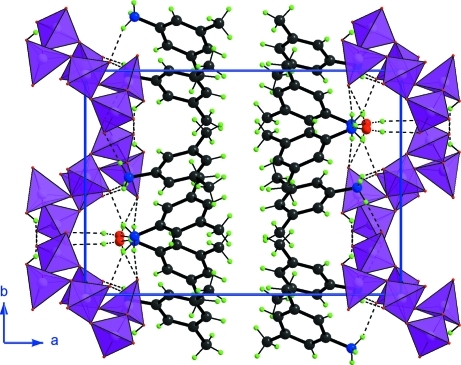
Projection of (I) along the c axis.

## supplementary crystallographic information

### Comment

Following investigations of Schülke and Kayser (Schulke, *et al.*, 1985)on the condensation-cyclization on LiH_2_PO_4_ into Li_6_P_6_O_18_, the crystal chemistry of cyclohexaphosphates developed rapidly. In the present investigation we report synthesis and crystal structure of a first cyclohexaphosphate acid, [3,5-(CH_3_)_2_C_6_H_3_NH_3_]_4_H_2_P_6_O_18_.2H_2_O, (I).

The title compound, is built up from H_2_P_6_O_18_^4-^ anion, four organic 3,5-xylidiniuium cations and two water molecules (Fig. 1). A half of the anion, two organic cations and a water molecule constitute the asymmetric unit of (I).

The atomic arrangement is a typical organization in layers as shows the figure 2. These corrugated layers are constituted of anions and water molecules that develop in the same way to plans (b,c) in *x* = 0. Charge compensation of these layers is achieved by the incorporation of the protonated 3,5-xylidinium cation in the interlayer spaces establishing H-bonds *via* their NH_3_ groups with H_2_P_6_O_18_ rings and water molecules. Inside such a structure, the phosphoric ring has an -1 internal symmetry. It develops around the inversion centers (0,0,0) and (0,1/2,1/2), so it is built up by only three independent tetrahedra. Among the P—O distances in PO_4_ tetrahedra, we can distinguish three different types. The longest ones correspond to the bridging oxygen atom, the intermediate one, corresponds to the P—OH bonding and the shortest, correspond to the external oxygen atoms. The calculated average values of the distortion indices (Baur, 1974) corresponding to the different angles and distances in the PO_4_ tetrahedra [DI (OPO) = 0.040; DI (PO) = 0.037; and DI (OO) = 0.016], show a pronounced distortion of the PO distances and OPO angles if compared to OO distances. So, the phosphate group can be considered as a rigid regular arrangement of oxygen atoms, with the phosphorus atom displaced from the gravity centre. It is worth noting that the strong H-bond between phosphoric rings (Table 1)(dO···O = 2.421 (2) Å < 2.73 Å) is never observed in cyclohexaphosphates.

With regards to the organic cation arrangement, these groups are in opposition, by creating thus a local invesion center. Interatomic bond lengths and angles of these groups spread within the respective ranges of 1.371 (3)–1.466 (2) Å and 118.2 (2)–122.1 (2)°. These values are similar to those obtained with the same isomers [Khederi, *et al.*, 2001, Rayes, *et al.*, 2004, Amri, *et al.*, 2008] The aromatic ring of the protonated used amine display an almost coplanar configuration with mean plane deviation of 0.000085 Å and 0.000245 Å. The interplanar distance between the aryl rings of the organic cations is in the vicinity of 4.00 Å, which is significantly longer than 3.80 Å for the π-π interaction (Janiak, 2000). The cohesion forces in this compound are assured by electrostatic interactions, van der Waals contacts and hydrogen bonds (O—H···O, N—H···O).

### Experimental

The title compound, [3,5-(CH_3_)_2_C_6_H_3_NH_3_]_4_H_2_P_6_O_18_.2H_2_O was synthesized by reaction of the cyclohexaphosphoric acid on 3,5-xylidine in an aqueous solution. The used acid was produced from a Li_6_P_6_O_18_ (Schulke *et al.*, 1985) solution by cation exchange on resins (Amberlite IR 120). The obtained H_6_P_6_O_18_ was added until the a pH between 1 and 2 in the final solution resulted. The same method of preparation was used for the synthesis of [3,5-(CH_3_)_2_C_6_H_3_NH_3_]_6_P_6_O_18_.6H_2_O, but in a less acidic medium (Khederi, *et al.*, 2001). Then this solution was slowly evaporated at room temperature for several days until the formation of transparent prisms of (I) were obtained.

### Crystal data

**Table:**

4C_8_H_12_N^+^·H_2_P_6_O_18_^4^^−^·2H_2_O	*F*(000) = 1048
*M**_r_* = 1000.61	*D*_x_ = 1.477 Mg m^−^^3^
Monoclinic, *P*2_1_/*c*	Mo *K*α radiation, λ = 0.71073 Å
Hall symbol: -P 2ybc	Cell parameters from 25 reflections
*a* = 17.254 (3) Å	θ = 6.3–10.1°
*b* = 11.763 (5) Å	µ = 0.32 mm^−^^1^
*c* = 11.556 (2) Å	*T* = 293 K
β = 106.41 (3)°	Prism, colourless
*V* = 2249.9 (11) Å^3^	0.35 × 0.20 × 0.01 mm
*Z* = 2	

### Data collection

**Table:**

Enraf–Nonius CAD-4 diffractometer	*R*_int_ = 0.039
Radiation source: Enraf Nonius FR590	θ_max_ = 35.0°, θ_min_ = 3.0°
graphite	*h* = 0→27
non–profiled ω scans	*k* = −18→0
10097 measured reflections	*l* = −18→17
9844 independent reflections	2 standard reflections every 120 min
5567 reflections with *I* > 2σ(*I*)	intensity decay: 11%

### Refinement

**Table:**

Refinement on *F*^2^	Primary atom site location: structure-invariant direct methods
Least-squares matrix: full	Secondary atom site location: difference Fourier map
*R*[*F*^2^ > 2σ(*F*^2^)] = 0.052	Hydrogen site location: inferred from neighbouring sites
*wR*(*F*^2^) = 0.141	H atoms treated by a mixture of independent and constrained refinement
*S* = 1.02	*w* = 1/[σ^2^(*F*_o_^2^) + (0.0664*P*)^2^ + 0.0078*P*] where *P* = (*F*_o_^2^ + 2*F*_c_^2^)/3
9844 reflections	(Δ/σ)_max_ = 0.001
295 parameters	Δρ_max_ = 0.50 e Å^−^^3^
3 restraints	Δρ_min_ = −0.51 e Å^−^^3^

### Special details

**Table:**

Geometry. All e.s.d.'s (except the e.s.d. in the dihedral angle between two l.s. planes) are estimated using the full covariance matrix. The cell e.s.d.'s are taken into account individually in the estimation of e.s.d.'s in distances, angles and torsion angles; correlations between e.s.d.'s in cell parameters are only used when they are defined by crystal symmetry. An approximate (isotropic) treatment of cell e.s.d.'s is used for estimating e.s.d.'s involving l.s. planes.
Refinement. Refinement of *F*^2^ against ALL reflections. The weighted *R*-factor *wR* and goodness of fit *S* are based on *F*^2^, conventional *R*-factors *R* are based on *F*, with *F* set to zero for negative *F*^2^. The threshold expression of *F*^2^ > σ(*F*^2^) is used only for calculating *R*-factors(gt) *etc*. and is not relevant to the choice of reflections for refinement. *R*-factors based on *F*^2^ are statistically about twice as large as those based on *F*, and *R*- factors based on ALL data will be even larger.

### Fractional atomic coordinates and isotropic or equivalent isotropic displacement parameters (Å^2^)

**Table:**

	*x*	*y*	*z*	*U*_iso_*/*U*_eq_	
P1	0.00136 (3)	0.52037 (4)	0.73695 (4)	0.02425 (10)	
P2	0.09276 (3)	0.68827 (4)	0.63729 (4)	0.02327 (10)	
P3	0.13602 (3)	0.55149 (4)	0.45087 (4)	0.02452 (10)	
O1	−0.04452 (9)	0.43770 (12)	0.62693 (12)	0.0358 (3)	
O2	−0.05542 (9)	0.55967 (13)	0.80125 (13)	0.0383 (3)	
O3	0.07729 (8)	0.46237 (12)	0.80005 (12)	0.0331 (3)	
O4	0.01792 (9)	0.62742 (15)	0.66154 (17)	0.0516 (5)	
O5	0.06005 (9)	0.77774 (12)	0.54796 (13)	0.0355 (3)	
O6	0.15290 (10)	0.71634 (14)	0.75342 (13)	0.0463 (4)	
O7	0.13336 (10)	0.58897 (13)	0.58091 (12)	0.0411 (4)	
O8	0.18549 (10)	0.63572 (15)	0.40517 (15)	0.0470 (4)	
H8	0.1563	0.6703	0.3476	0.071*	
O9	0.16580 (8)	0.43352 (11)	0.46188 (12)	0.0299 (3)	
O1W	0.10923 (9)	0.23683 (13)	0.55367 (13)	0.0350 (3)	
H2W	0.1207 (13)	0.3026 (12)	0.534 (2)	0.049 (8)*	
H1W	0.0587 (6)	0.226 (2)	0.535 (2)	0.051 (8)*	
N1	0.15651 (10)	0.26040 (14)	0.80220 (14)	0.0286 (3)	
H1A	0.1545	0.1974	0.8438	0.043*	
H1B	0.1444	0.2439	0.7240	0.043*	
H1C	0.1211	0.3107	0.8146	0.043*	
N2	0.13845 (10)	0.97750 (14)	0.54343 (15)	0.0330 (3)	
H2A	0.1291	1.0056	0.4692	0.050*	
H2B	0.1225	1.0275	0.5899	0.050*	
H2C	0.1111	0.9129	0.5408	0.050*	
C1	0.23806 (11)	0.30866 (16)	0.84216 (16)	0.0277 (3)	
C2	0.26029 (13)	0.38692 (18)	0.76896 (19)	0.0374 (5)	
H2	0.2242	0.4077	0.6958	0.045*	
C3	0.33672 (15)	0.4345 (2)	0.8052 (2)	0.0442 (5)	
C4	0.38869 (14)	0.4024 (2)	0.9153 (2)	0.0448 (5)	
H4	0.4401	0.4342	0.9397	0.054*	
C5	0.36648 (13)	0.3244 (2)	0.9902 (2)	0.0389 (5)	
C6	0.28937 (12)	0.27755 (18)	0.95214 (18)	0.0338 (4)	
H6	0.2726	0.2255	1.0007	0.041*	
C7	0.3613 (2)	0.5219 (3)	0.7259 (3)	0.0738 (10)	
H7A	0.3367	0.5936	0.7336	0.111*	
H7B	0.3438	0.4974	0.6434	0.111*	
H7C	0.4190	0.5301	0.7505	0.111*	
C8	0.42344 (16)	0.2895 (3)	1.1093 (2)	0.0611 (8)	
H8A	0.4552	0.2259	1.0973	0.092*	
H8B	0.3930	0.2683	1.1638	0.092*	
H8C	0.4584	0.3519	1.1428	0.092*	
C9	0.22502 (12)	0.95537 (16)	0.59349 (17)	0.0301 (4)	
C10	0.26548 (14)	0.99879 (18)	0.70482 (18)	0.0376 (5)	
H10	0.2387	1.0454	0.7460	0.045*	
C11	0.34615 (15)	0.9726 (2)	0.7550 (2)	0.0457 (5)	
C12	0.38358 (15)	0.9013 (2)	0.6916 (2)	0.0508 (6)	
H12	0.4376	0.8823	0.7253	0.061*	
C13	0.34264 (15)	0.8571 (2)	0.5790 (2)	0.0454 (5)	
C14	0.26241 (14)	0.88654 (19)	0.5298 (2)	0.0391 (5)	
H14	0.2340	0.8598	0.4540	0.047*	
C15	0.3918 (2)	1.0199 (3)	0.8766 (3)	0.0760 (10)	
H15A	0.4073	1.0970	0.8674	0.114*	
H15B	0.4392	0.9749	0.9101	0.114*	
H15C	0.3579	1.0179	0.9296	0.114*	
C16	0.3838 (2)	0.7769 (3)	0.5129 (3)	0.0766 (10)	
H16A	0.3464	0.7193	0.4731	0.115*	
H16B	0.4293	0.7419	0.5695	0.115*	
H16C	0.4021	0.8186	0.4542	0.115*	

### Atomic displacement parameters (Å^2^)

**Table:**

	*U*^11^	*U*^22^	*U*^33^	*U*^12^	*U*^13^	*U*^23^
P1	0.0263 (2)	0.0223 (2)	0.0234 (2)	0.00168 (17)	0.00594 (16)	0.00207 (16)
P2	0.0260 (2)	0.01997 (19)	0.02237 (19)	−0.00016 (16)	0.00439 (16)	−0.00186 (15)
P3	0.0315 (2)	0.0211 (2)	0.01944 (19)	0.00324 (17)	0.00459 (17)	−0.00015 (15)
O1	0.0347 (7)	0.0350 (7)	0.0313 (7)	0.0121 (6)	−0.0010 (6)	−0.0107 (6)
O2	0.0436 (8)	0.0418 (8)	0.0324 (7)	0.0089 (7)	0.0154 (6)	−0.0037 (6)
O3	0.0305 (7)	0.0324 (7)	0.0315 (7)	0.0082 (6)	0.0007 (5)	0.0033 (5)
O4	0.0324 (8)	0.0516 (10)	0.0708 (11)	0.0029 (7)	0.0147 (8)	0.0358 (9)
O5	0.0356 (7)	0.0252 (6)	0.0419 (8)	0.0001 (6)	0.0045 (6)	0.0094 (6)
O6	0.0525 (10)	0.0407 (9)	0.0335 (8)	0.0069 (7)	−0.0078 (7)	−0.0156 (6)
O7	0.0591 (10)	0.0392 (8)	0.0218 (6)	0.0212 (7)	0.0061 (6)	−0.0037 (6)
O8	0.0470 (9)	0.0448 (9)	0.0454 (9)	−0.0054 (8)	0.0066 (7)	0.0210 (7)
O9	0.0348 (7)	0.0230 (6)	0.0306 (6)	0.0049 (5)	0.0073 (5)	−0.0017 (5)
O1W	0.0338 (8)	0.0331 (8)	0.0359 (7)	−0.0025 (6)	0.0064 (6)	0.0061 (6)
N1	0.0291 (8)	0.0261 (7)	0.0294 (7)	−0.0014 (6)	0.0061 (6)	−0.0001 (6)
N2	0.0352 (9)	0.0279 (8)	0.0350 (8)	−0.0015 (7)	0.0085 (7)	0.0026 (7)
C1	0.0264 (8)	0.0258 (8)	0.0294 (8)	−0.0014 (7)	0.0055 (7)	−0.0015 (7)
C2	0.0392 (11)	0.0373 (11)	0.0323 (10)	−0.0051 (9)	0.0044 (8)	0.0045 (8)
C3	0.0442 (12)	0.0455 (13)	0.0428 (12)	−0.0135 (10)	0.0122 (10)	0.0040 (10)
C4	0.0312 (11)	0.0496 (13)	0.0508 (13)	−0.0097 (10)	0.0067 (10)	−0.0007 (11)
C5	0.0313 (10)	0.0422 (12)	0.0380 (11)	0.0008 (9)	0.0015 (8)	−0.0005 (9)
C6	0.0322 (10)	0.0350 (10)	0.0324 (9)	−0.0008 (8)	0.0059 (8)	0.0036 (8)
C7	0.071 (2)	0.080 (2)	0.0672 (19)	−0.0334 (17)	0.0143 (16)	0.0215 (16)
C8	0.0433 (14)	0.078 (2)	0.0486 (14)	−0.0039 (13)	−0.0086 (11)	0.0127 (13)
C9	0.0334 (9)	0.0254 (8)	0.0316 (9)	−0.0034 (7)	0.0091 (7)	0.0019 (7)
C10	0.0438 (12)	0.0372 (11)	0.0306 (9)	0.0016 (9)	0.0087 (9)	−0.0024 (8)
C11	0.0446 (12)	0.0513 (14)	0.0341 (11)	−0.0006 (11)	−0.0003 (9)	−0.0010 (10)
C12	0.0362 (12)	0.0537 (15)	0.0579 (15)	0.0039 (11)	0.0057 (11)	0.0023 (12)
C13	0.0438 (12)	0.0412 (12)	0.0558 (14)	−0.0026 (10)	0.0217 (11)	−0.0076 (11)
C14	0.0423 (12)	0.0380 (11)	0.0377 (10)	−0.0082 (9)	0.0124 (9)	−0.0095 (9)
C15	0.070 (2)	0.090 (2)	0.0484 (15)	0.0066 (18)	−0.0148 (14)	−0.0129 (16)
C16	0.0589 (18)	0.082 (2)	0.098 (3)	0.0113 (17)	0.0369 (18)	−0.028 (2)

### Geometric parameters (Å, °)

**Table:**

P1—O2	1.4619 (15)	C3—C7	1.515 (3)
P1—O3	1.4744 (14)	C4—C5	1.388 (3)
P1—O4	1.6024 (16)	C4—H4	0.9300
P1—O1	1.6187 (15)	C5—C6	1.392 (3)
P2—O5	1.4706 (15)	C5—C8	1.505 (3)
P2—O6	1.4832 (15)	C6—H6	0.9300
P2—O4	1.5692 (16)	C7—H7A	0.9600
P2—O7	1.5930 (15)	C7—H7B	0.9600
P3—O9	1.4728 (15)	C7—H7C	0.9600
P3—O8	1.4980 (16)	C8—H8A	0.9600
P3—O7	1.5790 (14)	C8—H8B	0.9600
P3—O1^i^	1.5859 (15)	C8—H8C	0.9600
O1—P3^i^	1.5859 (15)	C9—C14	1.371 (3)
O8—H8	0.8200	C9—C10	1.377 (3)
O1W—H2W	0.847 (9)	C10—C11	1.383 (3)
O1W—H1W	0.846 (9)	C10—H10	0.9300
N1—C1	1.466 (2)	C11—C12	1.387 (4)
N1—H1A	0.8900	C11—C15	1.510 (3)
N1—H1B	0.8900	C12—C13	1.393 (3)
N1—H1C	0.8900	C12—H12	0.9300
N2—C9	1.465 (3)	C13—C14	1.384 (3)
N2—H2A	0.8900	C13—C16	1.512 (4)
N2—H2B	0.8900	C14—H14	0.9300
N2—H2C	0.8900	C15—H15A	0.9600
C1—C2	1.376 (3)	C15—H15B	0.9600
C1—C6	1.377 (3)	C15—H15C	0.9600
C2—C3	1.384 (3)	C16—H16A	0.9600
C2—H2	0.9300	C16—H16B	0.9600
C3—C4	1.386 (3)	C16—H16C	0.9600
			
O2—P1—O3	121.63 (9)	C4—C5—C8	121.8 (2)
O2—P1—O4	106.02 (10)	C6—C5—C8	120.0 (2)
O3—P1—O4	111.25 (9)	C1—C6—C5	119.6 (2)
O2—P1—O1	109.87 (9)	C1—C6—H6	120.2
O3—P1—O1	106.24 (8)	C5—C6—H6	120.2
O4—P1—O1	99.66 (10)	C3—C7—H7A	109.5
O5—P2—O6	120.50 (10)	C3—C7—H7B	109.5
O5—P2—O4	106.25 (9)	H7A—C7—H7B	109.5
O6—P2—O4	109.92 (11)	C3—C7—H7C	109.5
O5—P2—O7	111.32 (9)	H7A—C7—H7C	109.5
O6—P2—O7	104.90 (9)	H7B—C7—H7C	109.5
O4—P2—O7	102.57 (10)	C5—C8—H8A	109.5
O9—P3—O8	115.72 (10)	C5—C8—H8B	109.5
O9—P3—O7	106.50 (8)	H8A—C8—H8B	109.5
O8—P3—O7	108.87 (10)	C5—C8—H8C	109.5
O9—P3—O1^i^	113.06 (8)	H8A—C8—H8C	109.5
O8—P3—O1^i^	108.80 (9)	H8B—C8—H8C	109.5
O7—P3—O1^i^	103.02 (9)	C14—C9—C10	121.9 (2)
P3^i^—O1—P1	125.79 (9)	C14—C9—N2	118.34 (18)
P2—O4—P1	137.54 (11)	C10—C9—N2	119.63 (18)
P3—O7—P2	136.74 (10)	C9—C10—C11	119.6 (2)
P3—O8—H8	109.5	C9—C10—H10	120.2
H2W—O1W—H1W	111.4 (19)	C11—C10—H10	120.2
C1—N1—H1A	109.5	C10—C11—C12	118.5 (2)
C1—N1—H1B	109.5	C10—C11—C15	120.4 (2)
H1A—N1—H1B	109.5	C12—C11—C15	121.1 (2)
C1—N1—H1C	109.5	C11—C12—C13	122.0 (2)
H1A—N1—H1C	109.5	C11—C12—H12	119.0
H1B—N1—H1C	109.5	C13—C12—H12	119.0
C9—N2—H2A	109.5	C14—C13—C12	118.4 (2)
C9—N2—H2B	109.5	C14—C13—C16	120.5 (2)
H2A—N2—H2B	109.5	C12—C13—C16	121.2 (2)
C9—N2—H2C	109.5	C9—C14—C13	119.6 (2)
H2A—N2—H2C	109.5	C9—C14—H14	120.2
H2B—N2—H2C	109.5	C13—C14—H14	120.2
C2—C1—C6	121.79 (18)	C11—C15—H15A	109.5
C2—C1—N1	118.35 (17)	C11—C15—H15B	109.5
C6—C1—N1	119.83 (17)	H15A—C15—H15B	109.5
C1—C2—C3	119.47 (19)	C11—C15—H15C	109.5
C1—C2—H2	120.3	H15A—C15—H15C	109.5
C3—C2—H2	120.3	H15B—C15—H15C	109.5
C2—C3—C4	118.8 (2)	C13—C16—H16A	109.5
C2—C3—C7	119.8 (2)	C13—C16—H16B	109.5
C4—C3—C7	121.4 (2)	H16A—C16—H16B	109.5
C3—C4—C5	122.1 (2)	C13—C16—H16C	109.5
C3—C4—H4	118.9	H16A—C16—H16C	109.5
C5—C4—H4	118.9	H16B—C16—H16C	109.5
C4—C5—C6	118.2 (2)		

Symmetry codes: (i) −*x*, −*y*+1, −*z*+1.

### Hydrogen-bond geometry (Å, °)

**Table:**

*D*—H···*A*	*D*—H	H···*A*	*D*···*A*	*D*—H···*A*
O8—H8···O6^ii^	0.82	1.71	2.421 (2)	144
O1W—H2W···O9	0.85 (1)	2.01 (1)	2.831 (2)	164 (2)
O1W—H1W···O5^i^	0.85 (1)	2.00 (1)	2.829 (2)	165 (2)
N1—H1A···O9^iii^	0.89	2.03	2.910 (2)	170
N1—H1B···O1W	0.89	1.89	2.769 (2)	169
N1—H1C···O3	0.89	1.93	2.738 (2)	151
N2—H2A···O3^ii^	0.89	1.94	2.801 (2)	161
N2—H2B···O2^iv^	0.89	1.97	2.768 (2)	148
N2—H2C···O5	0.89	1.83	2.719 (2)	175

Symmetry codes: (ii) *x*, −*y*+3/2, *z*−1/2; (i) −*x*, −*y*+1, −*z*+1; (iii) *x*, −*y*+1/2, *z*+1/2; (iv) −*x*, *y*+1/2, −*z*+3/2.
